# Trends and emerging frontiers of mesenchymal stem cells in intervertebral disc degeneration: a bibliometric analysis (2000–2024)

**DOI:** 10.3389/fmed.2025.1601806

**Published:** 2025-06-25

**Authors:** Jian Zhao, Shuwen Li, Jie Cheng, Xiang Xu, Ming Bai, Yingnan Yu, Meixia Liu, Heping Yin, Yimin Wu

**Affiliations:** Second Affiliated Hospital of Inner Mongolia Medical University, Hohhot, China

**Keywords:** intervertebral disc degeneration, mesenchymal stem cells, regenerative medicine, bibliometric analysis, CiteSpace, VOSviewer

## Abstract

**Background:**

Intervertebral disc degeneration (IVDD) is a major global cause of disability, and mesenchymal stem cell (MSC) therapy offers a promising regenerative solution by targeting the root causes of degeneration, unlike conventional symptom-focused treatments. This bibliometric analysis explores trends and emerging research areas in MSC applications for IVDD.

**Methods:**

A comprehensive literature search was conducted in the Web of Science Core Collection database, covering publications from 2000 to 2024. Bibliometric and visualized analysis was performed using VOSviewers, CiteSpace and the R package “Bibliometrix.”

**Results:**

This bibliometric analysis reviewed 931 articles, revealing an overall upward trend in publication activity. Leading authors included Sakai Daisuke, Grad Sibylle, and Alini Mauro. China and the USA led in publication volume and citation counts, while the United Kingdom achieved the highest average citations per publication. The University of Pennsylvania and Zhejiang University were the most productive institutions. Key journals included *Biomaterials*, *Spine*, and *Tissue Engineering Part A*. Earlier core keywords primarily focused on foundational concepts such as “gene expression,” “articular cartilage,” “anulus fibrosus” and “extracellular matrix.” Recent keyword bursts include “activation,” “autophagy,” “extracellular vesicles,” “apoptosis,” “exosome,” and “oxidative stress.”

**Conclusion:**

This bibliometric analysis revealed key research focuses on foundational biological mechanisms, translational applications, and addressing specific challenges in the use of MSC for IVDD. Future research is likely to focus on optimizing MSC functionality, developing cell-free therapies such as extracellular vesicles, and targeting the molecular mechanisms involved in disc degeneration and regeneration.

## Background

Intervertebral disc degeneration (IVDD) is an age-related condition marked by the progressive loss of hydration, extracellular matrix degradation, and impaired mechanical properties of the disc, leading to chronic lower back pain, radiculopathy, and reduced mobility (1). Affecting over 80% of adults during their lifetime, IVDD is a leading cause of disability worldwide, particularly in individuals over 50 (2). Current treatments, including physical therapy, pain management, and surgery, focus on symptom relief rather than reversing degeneration, often failing to address the underlying pathology and resulting in limited long-term success (3).

Regenerative medicine, particularly mesenchymal stem cell (MSC) therapy, offers a promising alternative by targeting the root causes of IVDD (4). MSC, capable of differentiating into key cell types, can restore extracellular matrix integrity, improve disc hydration, and reduce inflammation (5). Preclinical and clinical studies have shown that MSC transplantation alleviates pain and promotes disc regeneration, with advancements such as bioengineered scaffolds and gene-editing techniques further enhancing therapeutic potential (6). Despite these developments, challenges remain, including optimizing cell delivery, ensuring long-term cell viability, and understanding repair mechanisms (7). Over the past several decades, numerous solving strategies have been proposed and implemented: (1) explore the repair mechanisms and identify more targets that promote differentiation; (2) improve the harsh microenvironment to provide a better living environment for loaded MSC; (3) establish standard methods that can induce and differentiate stem cells from different sources more efficiently and stably; (4) optimize the performance of MSC carrier to avoid secondary damage during implantation and enhance the repair ability (8).

To trace the evolution of this field, bibliometric analysis serves as a powerful tool for mapping publication trends, identifying influential studies, and uncovering collaborative networks (9). Existing bibliometric studies have explored MSC-derived exosomes in acute lung injury and acute respiratory distress syndrome (10), as well as global research trends on adipose-derived mesenchymal stem cells (11). However, no bibliometric analysis has specifically addressed the applications of MSC in IVDD. This study seeks to bridge this gap by examining MSC-related research in IVDD, explore key contributors and trends to inform future advancements in regenerative therapies.

## Methods

### Literature search and selection

A comprehensive literature search was conducted using the Web of Science Core Collection (WoSCC), a renowned multidisciplinary database for high-quality scientific research indexing (12). The search formula was carefully designed based on relevant prior studies (13–15) and tailored to the research focus: (TS = (“intervertebral disc*” OR “intervertebral disk*” OR “Disc*, Intervertebral” OR “Disk*, Intervertebral” OR “degenerative disc disease*”)) AND TS = (“mesenchymal stem cell*” OR “mesenchymal stromal cell*” OR “MSC” OR “MSCs”). The search covered English-language articles published between January 1, 2000, and September 3, 2024. Various document types, including review articles, meeting abstracts, editorial materials, proceeding papers, corrections, letters, and non-English publications, were excluded during the screening process.

### Statistical analysis and visualization

For the analysis and visualization of bibliometric data, we employed three powerful tools: VOSviewer (version 1.6.20), CiteSpace (version 6.3. R1), and R package “Bibliometrix” (version 4.4.1).

VOSviewer was used to map collaboration networks, including institutional partnerships, author co-authorship, and keyword co-occurrence. Its intuitive and interactive visualizations provided insights into complex relationships between researchers, institutions, and key themes (16).

CiteSpace was utilized primarily for detecting keyword bursts, identifying research hotspots, and analyzing temporal shifts in academic focus (17). The time span for analysis was set from January 2000 to September 2024. The time slicing was configured to span one-year intervals, and node types were set to keywords. When nodes were designated as keywords, the threshold was set to the top N per slice (*N* = 5), and pruning was conducted using the Pathfinder network merging algorithm.

The R package “Bibliometrix” was utilized for trend mapping and ranking analyses, enabling the tracking of publication and citation patterns among authors, institutions, and countries. This tool further facilitated the generation of trend charts and longitudinal analyses concerning the evolution of the research field (18). Various bibliometric indices, including the h-index, g-index, and m-index, were employed to assess the academic impact of authors and journals. The h-index measures both productivity and citation influence, reflecting the number of publications with at least h citations (19). The g-index assigns more weight to highly cited works, while the m-index normalizes the h-index by an author’s career length, offering a temporal perspective (20). These metrics were derived from data exported from the WoSCC database. Journal quality and impact were assessed using Journal Citation Reports (JCR) quartile rankings and Impact Factor (IF). The JCR quartiles (Q1–Q4) categorize journals by their relative IF within specific disciplines, serving as benchmarks for academic influence.

## Results

### An overview of publications

**Figure 1:**
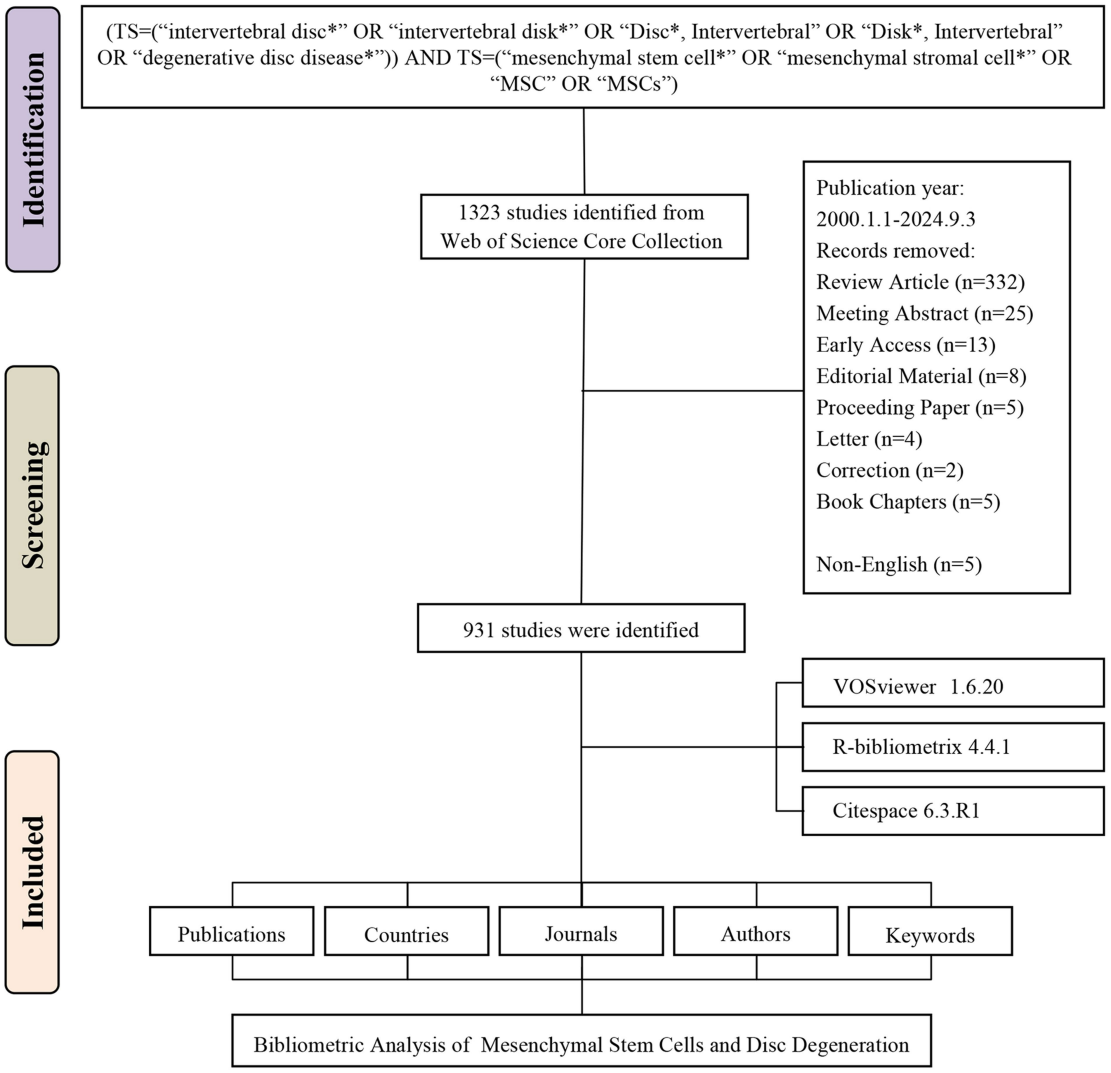
Flowchart of the literature screening process.

**Figure 2:**
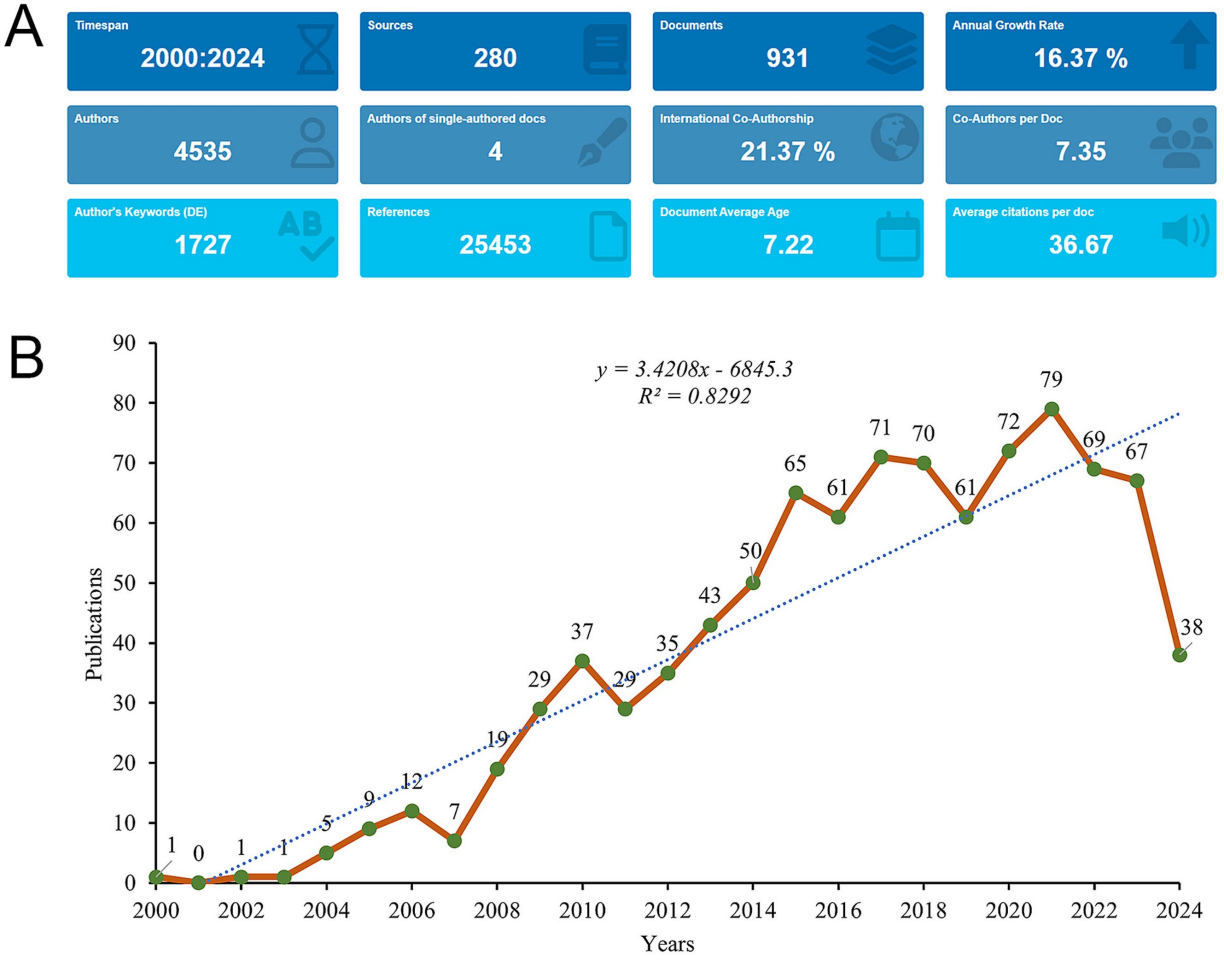
Analysis of general information. **(A)** Summary Information of the included studies. **(B)** Annual number of publications on mesenchymal stem cells in IVDD from 2000 to 2024.

A total of 1,323 records were initially retrieved in WoSCC database. After the removal of review, meeting abstract, early access, editorial material, etc., 931 studies published between January 1, 2001 and September 3, 2024 were included for analysis (Figure 1). The studies were published in 280 different journals, referencing a total of 25,453 citations (Figure 2A). From 2000 to 2021, publication activity showed an overall upward trend, although fluctuations were evident throughout the period, and the highest number of annual publications in the dataset occurred in 2021, with 79 publications. Besides, a noticeable decline was observed in the number of publications starting in 2022 (Figure 2B).

### Analysis of countries

**Figure 3:**
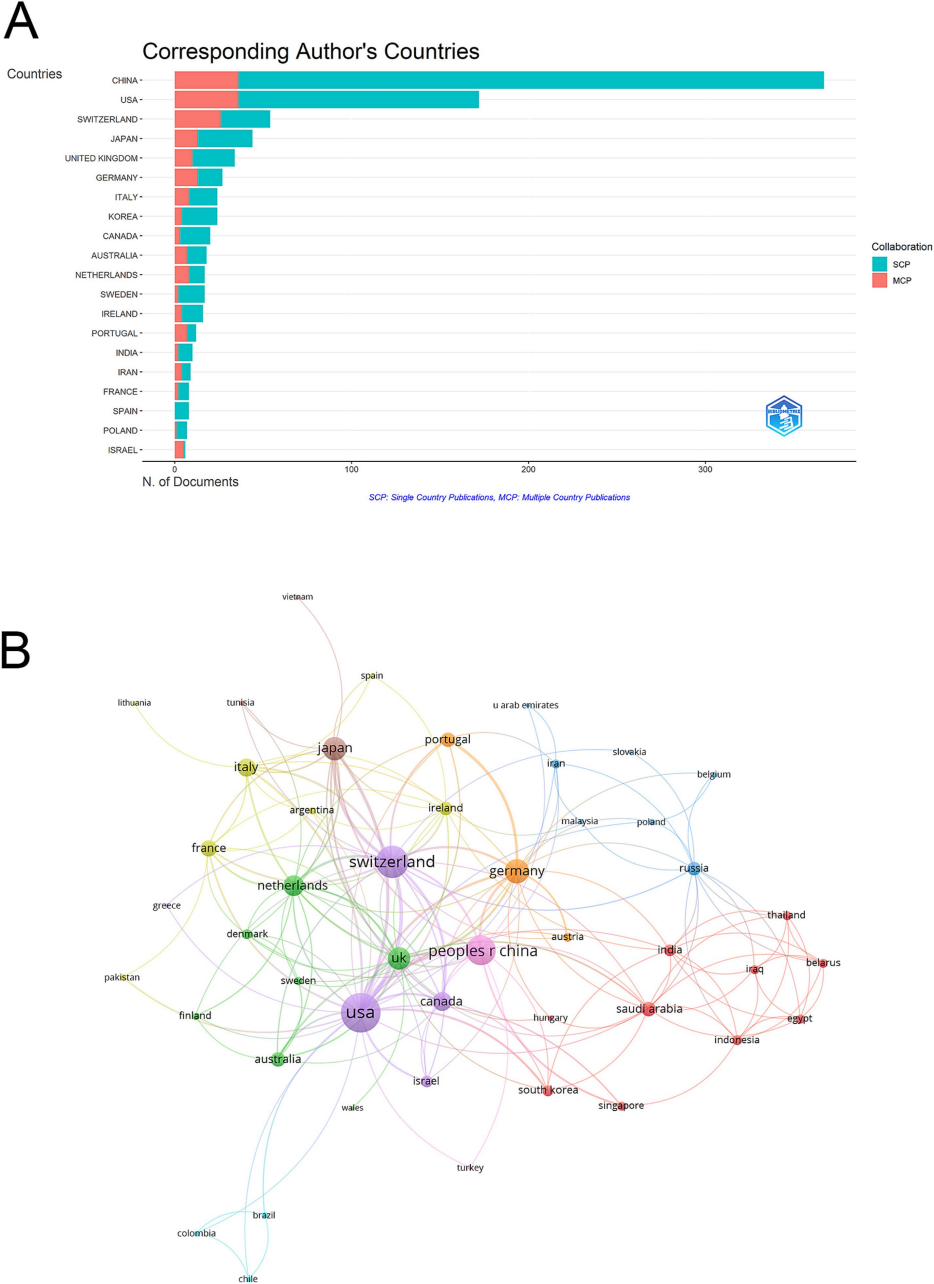
Analysis of countries. **(A)** Distribution of corresponding author’s publications by country. The number of publications attributed to corresponding authors from different countries, distinguishing between single country publications (SCP) and multiple country publications (MCP). **(B)** Visualization map depicting the collaboration among different countries. The collaborative relationships between countries, with nodes representing countries, the size of nodes indicating publication count, and the thickness of links showing the strength of co-authorship collaborations.

In the top 10 countries by publication volume, China ranked first with 367 articles, followed by the USA (172) and Switzerland (54). In terms of total citations, China also led with 9,147 citations, followed closely by the USA with 8,826 citations, while Japan ranked third with 3,083 citations. However, the United Kingdom recorded the highest average citations per publication at 73.1, followed by Japan (70.1) and the USA (51.3) (Table 1). Switzerland stood out with a high MCP Ratio (0.481) and ranked third in total publications and fifth in total citations (2,239). Notably, Spain, with only 8 articles, achieved an exceptional average citation rate of 101.9, showcasing its high impact despite limited output (Table 1 and Figure 3A).

**Table 1:** Publication and citation profiles of leading countries.

Country	Articles	Freq	SCP	MCP	MCP_Ratio	TP	TP_rank	TC	TC_rank	Average citations
China	367	0.394	331	36	0.098	1,117	1	9,147	1	24.9
USA	172	0.185	136	36	0.209	728	2	8,826	2	51.3
Switzerland	54	0.058	28	26	0.481	199	3	2,239	5	41.5
Japan	44	0.047	31	13	0.295	141	4	3,083	3	70.1
United Kingdom	34	0.037	24	10	0.294	102	6	2,486	4	73.1
Germany	27	0.029	14	13	0.481	119	5	1,000	6	37
Italy	24	0.026	16	8	0.333	97	8	855	7	35.6
Korea	24	0.026	20	4	0.167	98	7	810	9	33.8
Canada	20	0.021	17	3	0.15	89	9	761	10	38
Australia	18	0.019	11	7	0.389	86	10	697	11	38.7
Netherlands	17	0.018	9	8	0.471	68	12	374	15	22
Sweden	17	0.018	15	2	0.118	64	15	461	13	27.1
Ireland	16	0.017	12	4	0.25	67	13	495	12	30.9
Portugal	12	0.013	5	7	0.583	64	14	257	17	21.4
India	10	0.011	8	2	0.2	25	21	442	14	44.2
Iran	9	0.01	5	4	0.444	39	17	240	18	26.7
France	8	0.009	6	2	0.25	68	11	221	19	27.6
Spain	8	0.009	8	0	0	42	16	815	8	101.9
Poland	7	0.008	6	1	0.143	33	19	88	22	12.6
Israel	6	0.006	1	5	0.833	15	22	266	16	44.3

Articles: Publications of Corresponding Authors only. Freq: Frequence of Total Publications. MCP_Ratio: Proportion of Multiple Country Publications. TP: Total Publications. TP_rank: Rank of Total Publications. TC: Total Citations. TC_rank: Rank of Total Citations. Average Citations: The average number of citations per publication.

Among the 50 countries engaged in international collaborations with at least one published article, the USA led with the highest number of collaborations (total link strength = 128), followed by Switzerland (total link strength = 86) and China (total link strength = 72) (Figure 3B). In the country collaboration network, countries are grouped into color-coded clusters based on their co-authorship patterns. The purple cluster is centered around the USA and includes countries such as Switzerland, Canada, and Israel, indicating a strong international collaboration network. The green cluster comprises countries like the UK, Netherlands, Australia, Finland, Sweden, and Denmark, mainly representing Western European collaborations. The red cluster includes Saudi Arabia, Iraq, and other Asian countries, reflecting close regional cooperation. The yellow cluster consists of France, Italy, and Ireland. The blue cluster features countries such as Russia, Poland, and several Eastern European and Southeast Asian countries. These clusters illustrate distinct international collaborative groups in MSC research related to IVDD (Figure 3B).

### Analysis of institutions

**Figure 4:**
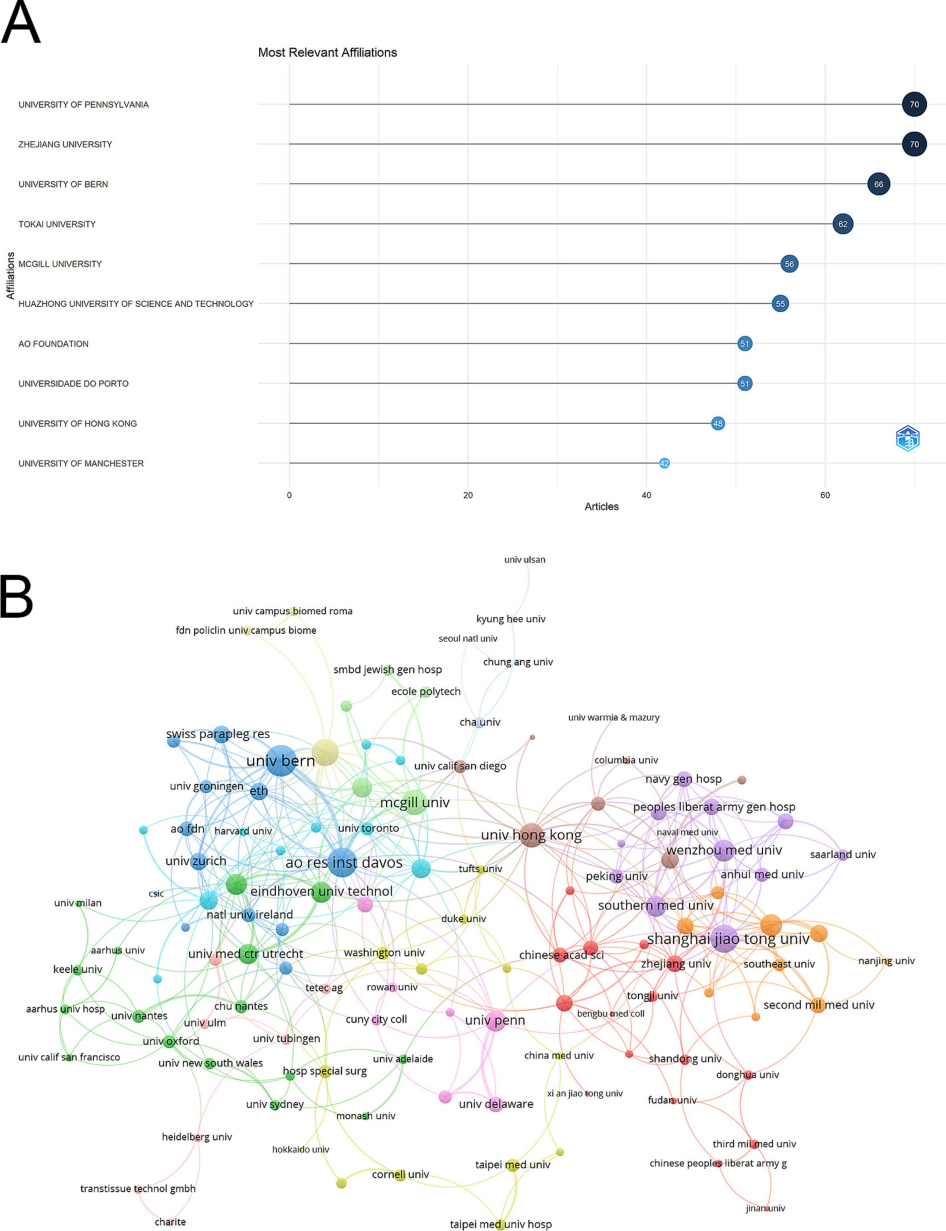
Analysis of institutions. **(A)** Top ten institutions by article count and rank. The circle size shows the article count, with darker shades indicating higher ranks. **(B)** Visualization map depicting the collaboration among different institutions. Nodes represent institutions, with size indicating publication count. Links represent co-authorships, with thickness showing collaboration strength. Colors indicate different research clusters. Total link strength in collaboration networks measures the frequency of co-authorship between institutions, indicating the level of collaborative research.

The University of Pennsylvania and Zhejiang University were the leading institutions, each publishing 70 articles. They were closely followed by the University of Bern with 66 articles and Tokai University with 62. Other significant contributors included McGill University (56 articles), Huazhong University of Science and Technology (55 articles), AO Foundation (51 articles), Universidade do Porto (51 articles), the University of Hong Kong (48 articles), and the University of Manchester with 42 articles (Figure 4A). Among the 139 institutions involved in international collaborations with at least four articles, the University of Bern led with the highest number of collaborations (total link strength = 58), followed by the AO Research Institute Davos (total link strength = 51) and Shanghai Jiao Tong University (total link strength = 45) (Figure 4B).

### Analysis of journals

An analysis of several bibliometric indicators for the top 20 most influential journals revealed that these journals published 404 papers, accounting for 43.4% of all the retrieved publications (Table 2). *Biomaterials* ranked first with an H-index of 26 and 29 publications, followed by *Spine* (H-index: 26) with 37 publications, and *Tissue Engineering Part A* (H-index: 22) with 39 publications. Regarding total citations, *Spine* dominated with 6,115 citations, while *Biomaterials* (1,842 citations) and the *European Spine Journal* (1,568 citations) ranked second and third, respectively. *Biomaterials* distinguished itself further with the highest IF of 12.8.

**Table 2:** Bibliometric indicators of high-impact journals.

Journal	H_index	G_index	M_index	IF 2023	JCR 2023	TP	TP_rank	TC	TC_rank	PY_start
Biomaterials	26	29	1.182	12.8	1	29	4	1842	2	2003
Spine	26	37	1.238	2.6	1	37	2	6,115	1	2004
Tissue Engineering Part A	22	37	1.294	3.5	2	39	1	824	7	2008
Journal of Orthopaedic Research	21	31	1.105	2.1	2	31	3	1,260	4	2006
Acta Biomaterialia	19	26	1.357	9.4	1	26	6	639	10	2011
European Cells & Materials	19	29	1.118	3.2	1	29	5	547	11	2008
Spine Journal	18	26	1.2	4.9	1	26	7	1,146	5	2010
Arthritis Research & Therapy	14	15	0.824	4.4	1	15	13	918	6	2008
European Spine Journal	14	20	0.933	2.6	1	20	9	1,568	3	2010
Stem Cell Research & Therapy	14	20	0.933	7.1	1	20	10	460	15	2010
PLoS One	13	16	0.929	2.9	1	16	12	547	12	2011
Stem Cells International	13	20	1.083	3.8	2	21	8	360	21	2013
Connective Tissue Research	11	13	0.55	2.8	1	13	15	234	38	2005
Journal of Tissue Engineering and Regenerative Medicine	11	12	0.688	3.1	2	12	17	392	17	2009
International Journal of Molecular Sciences	10	15	1	4.9	1	19	11	279	32	2015
Oxidative Medicine and Cellular Longevity	9	10	1.286	NA	NA	10	19	149	56	2018
Scientific Reports	9	10	0.9	3.8	1	10	20	289	30	2015
Current Stem Cell Research & Therapy	8	9	0.667	2.1	4	9	21	131	65	2013
Join spine	8	13	1.143	3.4	1	13	16	286	31	2018
Stem cells and development	8	9	0.667	2.5	2	9	22	345	23	2013

H_index: The h-index of the journal, which measures both the productivity and citation impact of the publications. G_index: The g-index of the journal, which gives more weight to highly-cited articles. M_index: The m-index of the journal, which is the h-index divided by the number of years since the first published paper. TP: Total Publications. TP_rank: Rank of Total Publications. TC: Total Citations. TC_rank: Rank of Total Citations. PY_start: Publication Year Start, indicating the year the journal started publication. IF 2023: Impact Factor in 2023, indicating the average number of citations to recent articles published in the journal. JCR 2023: The quartile ranking of the journal in the Journal Citation Reports in 2023, indicating the journal’s ranking relative to others in the same field (Q1: top 25%, Q2: 25–50%, Q3: 50–75%, Q4: bottom 25%).

**Figure 5:**
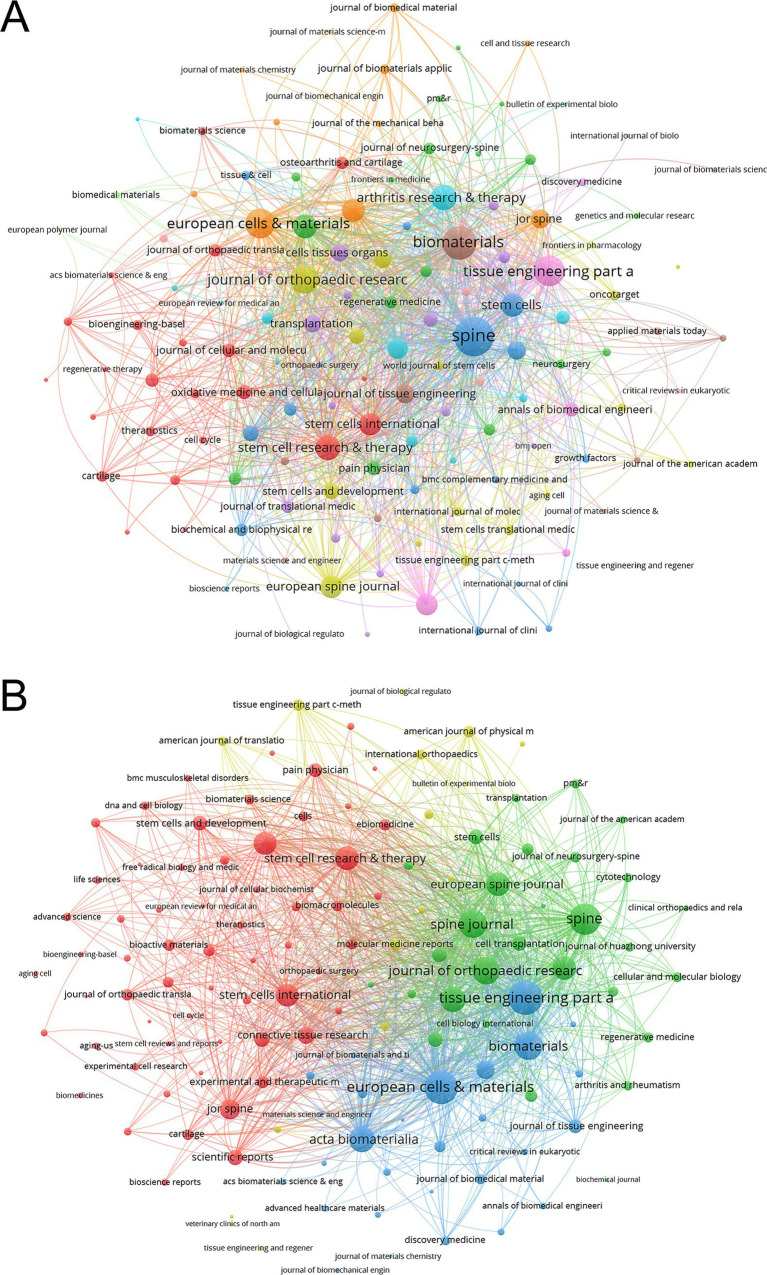
Analysis of journals. **(A)** Co-occurrence Network of Journals. The frequency with which journals are cited together within the same articles reflects thematic or topical connections between the research they publish. **(B)** Coupling Network of Journals. The extent to which journals are linked is based on common references cited in their articles, indicating a shared intellectual foundation or research focus.

The co-occurrence networks of journals in MSC and disc degeneration research featured 136 journals with at least two occurrences. *Spine* (971), *Biomaterials* (671), and *Tissue Engineering Part A* (533) stood out with the highest total link strength, indicating frequent co-citation in scholarly articles and strong thematic connections between their research (Figure 5A). In the coupling networks, which measure the degree of shared references among journals, 136 journals with at least two shared references were identified. *Tissue Engineering Part A* (28,318), *European Cells & Materials* (27,425), and *Spine* (23,736) had the highest link strength, reflecting a substantial overlap in their referenced literature and a shared research foundation (Figure 5B). Furthermore, the journals were grouped into distinct color-coded clusters based on citation coupling relationships. The green cluster includes journals such as *Spine*, *European Spine Journal*, and *Tissue Engineering Part A*, which exhibit strong mutual citation links. The red cluster consists of journals like *Stem Cell Research & Therapy,* and *Stem Cells International*, indicating another close citation network. The blue cluster includes *Biomaterials*, *European Cells & Materials, Acta Biomaterialia, Journal of Tissue Engineering*, and related journals. The yellow cluster comprises a smaller number of journals with relatively fewer citation links but still forms a discernible subgroup. These clusters reveal the citation coupling patterns and close citation relationships among journals within the field (Figure 5B).

### Analysis of authors

**Figure 6:**
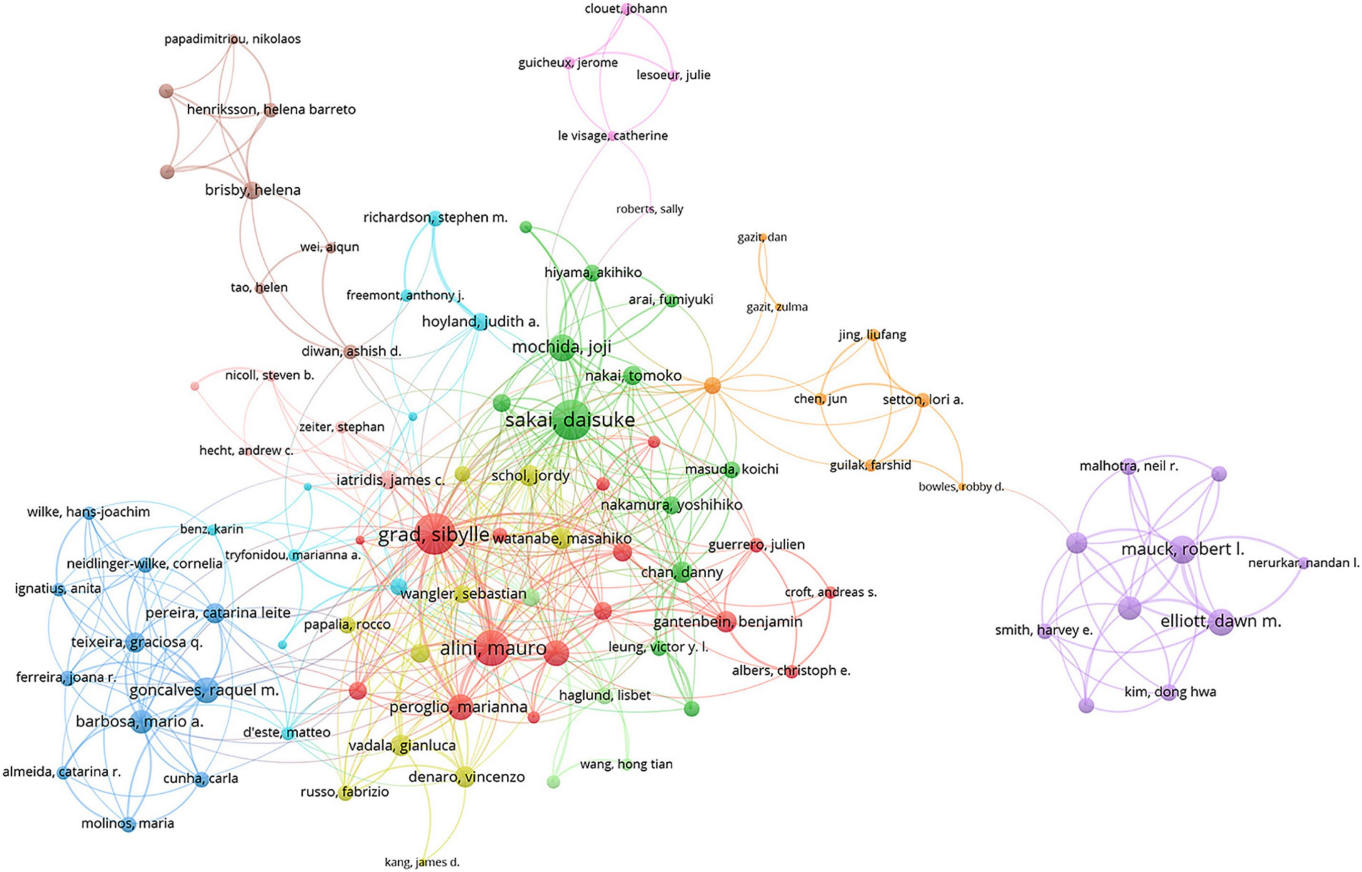
Visualization map depicting the collaboration among different authors. Nodes represent authors, with size indicating publication count. Links represent co-authorships, with thickness showing collaboration strength. Colors indicate different research clusters. Total link strength in collaboration networks measures the frequency of co-authorship between authors, indicating the level of collaborative research.

Leading in both H-index and total publications, Sakai Daisuke (H-index: 20, 29 publications) was followed by Grad Sibylle (H-index: 20, 27 publications) and Alini Mauro (H-index: 19, 21 publications). For total citations, Hoyland Judith A. ranked highest with 1,991 citations, while Sakai Daisuke (1,989 citations) and Grad Sibylle (1,806 citations) closely followed (Table 3). Of the 272 authors with at least four articles in international collaborations, Grad Sibylle led with the most collaborations (total link strength = 128), followed by Sakai Daisuke (total link strength = 120) and Alini Mauro (total link strength = 97) (Figure 6). The color-coded clusters in the network map represent co-authorship groups formed based on the frequency of collaboration. While the map itself does not explicitly indicate specific research topics, the core authors within each cluster offer valuable insights into the probable research focuses. For instance, the red cluster, predominantly featuring Grad Sibylle and Alini Mauro, is likely centered on intervertebral disc regeneration therapies. The green cluster, with key contributors such as Sakai Daisuke and Mochida Joji, appears to focus on cell-based therapies and the molecular mechanisms underlying disc degeneration. The blue cluster, including Wilke Hans-Joachim and Neidlinger-Wilke Cornelia, seems to concentrate on disc degeneration repair and biomarker exploration. Lastly, the orange cluster, led by Setton Lori A. and Bowles Robby D., is likely associated with matrix biology and biomaterial development for tissue repair. These interpretations are inferred from the primary research interests of the leading authors within each group (Figure 6).

**Table 3:** Publication and citation profiles of high-impact authors.

Author	h_index	g-index	m-index	PY_start	TP	TP_Frac	TP_rank	TC	TC_rank
Grad Sibylle	20	27	1.176	2008	27	3.58	2	1806	3
Sakai Daisuke	20	29	1.111	2007	29	4.07	1	1989	2
Alini Mauro	19	21	1.118	2008	21	2.99	3	1,452	5
Mauck Robert L.	15	17	0.938	2009	17	2.8	6	1,037	8
Elliott Dawn M.	14	16	0.875	2009	16	2.65	8	1,000	9
Shao Zengwu	14	17	1.077	2012	17	2.07	6	468	14
Hoyland Judith A.	13	19	0.684	2006	19	3.73	4	1991	1
Mochida Joji	13	14	0.722	2007	14	2.08	10	1,278	6
Benneker Lorin M.	12	13	0.857	2011	13	1.96	16	722	10
Iatridis James C.	12	13	0.706	2008	13	2	16	1,243	7
Li Hao	12	16	0.923	2012	16	2.11	8	489	13
Richardson Stephen M.	12	14	0.632	2006	14	2.71	10	1,611	4
Wang Feng	12	13	1.2	2015	13	1.61	16	352	19
Liang Chengzhen	11	14	0.846	2012	14	1.58	10	421	16
Peroglio Marianna	11	12	0.846	2012	12	1.84	19	493	12
Chen Qixin	10	14	0.769	2012	14	1.63	10	403	18
Li Fangcai	10	14	0.769	2012	14	1.61	10	410	17
Ruan Dike	10	18	0.769	2012	18	2.39	5	327	20
Zhou Xiaopeng	10	14	0.769	2012	14	1.61	10	455	15
Zhou Yue	10	11	0.714	2011	11	1.76	20	522	11

H_index: The h-index of the journal, which measures both the productivity and citation impact of the publications. g_index: The g-index of the journal, which gives more weight to highly-cited articles. m_index: The m-index of the journal, which is the h-index divided by the number of years since the first published paper. TP: Total Publications. TP_rank: Rank of Total Publications. TC: Total Citations. TC_rank: Rank of Total Citations. Average Citations: The average number of citations per publication. PY_start: Publication Year Start, indicating the year the journal started publication.

### Analysis of publications

The most cited article in this field, titled “The Achilles’ heel of senescent cells: from transcriptome to senolytic drugs,” was published in *Aging Cell* (IF = 8.0) in 2015 and has accumulated 1,413 citations (21). The second most cited article, “Exhaustion of nucleus pulposus progenitor cells with ageing and degeneration of the intervertebral disc,” was published in *Nature Communications* (IF = 14.7) in 2012, garnering 353 citations (22). The third most cited article, titled “Intervertebral disc repair by autologous mesenchymal bone marrow cells: a pilot study,” appeared in *Transplantation* (IF = 5.3) in 2011 and also received 353 citations (23).

### Analysis of keywords

The VOSviewer analysis identified 127 keywords with a minimum of 10 occurrences. Key terms such as “regeneration,” “differentiation,” “*in vitro*,” “*in vivo*,” “degeneration,” “repair,” and “low-back pain” appeared as larger nodes, signifying their high frequency and central importance in the research network. Keywords tied to major themes, including “transplantation,” “stromal cells,” and “gene expression,” underscoring significant research interest in these areas.

**Figure 7:**
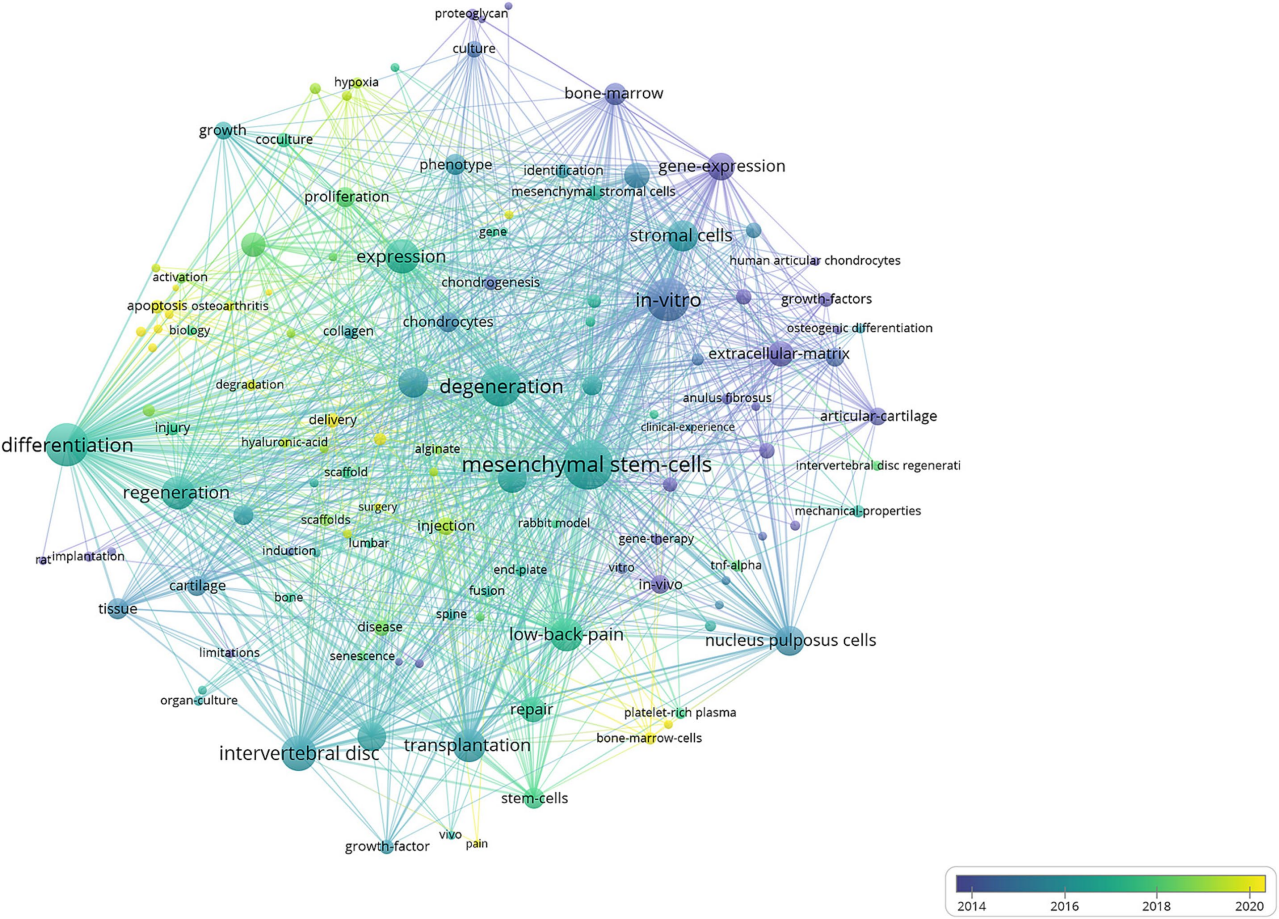
Visual analysis of keyword co-occurrence network analysis. Each node represents a keyword, with size indicating its frequency of occurrence. Links between nodes represent co-occurrence in the same documents, with thicker lines showing stronger associations. Colors reflect the average publication year of the articles, as indicated by the color gradient at the bottom right. The transition from purple to green to yellow represents the timeline of keywords, with purple indicating older terms and yellow representing the most recent ones.

Temporal evolution of research themes was visualized through a time-overlay network map (Figure 7), demonstrating distinct chronological patterns. Early-stage research (2014, represented by purple nodes) focused on foundational terms such as “gene expression,” “articular cartilage,” “anulus fibrosus,” and “extracellular matrix.” Mid-phase investigations (2016–2018, represented by green nodes) showed increased emphasis on regenerative potentials of MSC on intervertebral disc, particularly “regeneration,” “chondrocytes,” and “collagen.” Recent advancements (2020, represented by yellow nodes) highlighted emerging priorities in researches on development of delivery system and resources of MSC, as evidenced by high-frequency terms like “delivery,” and “bone-marrow-cells.” This trend reflected a gradual shift in research priorities towards optimizing MSC applications.

### Analysis of burst keywords

**Figure 8:**
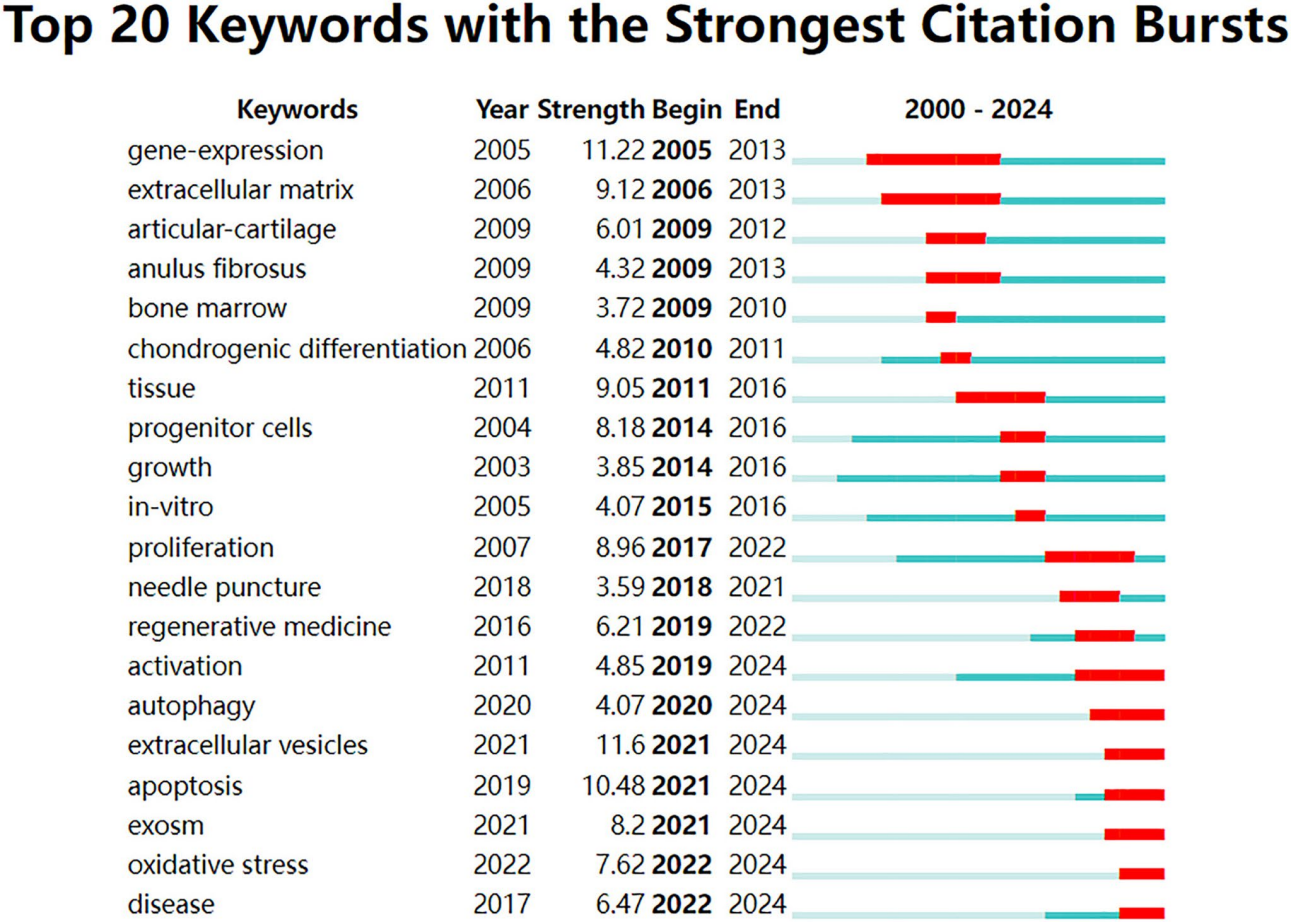
Top 20 keywords with the strongest citation bursts from 2000 to 2024. The blue lines represent the period, and the red lines indicate the burst periods of the keywords.

The analysis of the top 20 keywords with the strongest citation bursts from 2000 to 2024 revealed shifting research trends. The keyword with the highest burst strength was “extracellular vesicles” (11.6, 2021–2024), followed by “gene-expression” (11.22, 2005–2013). Earlier bursts highlighted topics like “articular-cartilage” (2009–2012), “anulus fibrosus” (2009–2013), and “bone marrow” (2009–2010). In contrast, recent bursts extending through 2024 focus on emerging areas such as “activation” (2019–2024), “autophagy” (2020–2024), “extracellular vesicles” (2021–2024), “apoptosis” (2021–2024), “exosome” (2021–2024), “oxidative stress” (2022–2024), and “disease” (2022–2024) (Figure 8).

## Discussion

This bibliometric analysis of 931 publications reveals an overall upward trend in research activity and provides an overview of the field. Geographical analysis revealed that MSC research in IVDD is predominantly led by China, the USA and Switzerland, which may be due to heavy health burden of low back pain (LBP), as the leading outcome of IVDD. Global Burden of Disease study showed that the USA and Switzerland had the highest age-standardized prevalence estimates of LBP (24). In the USA alone, the total costs of LBP exceed $100 billion per year (25). On the other hand, in China, numerous studies focused on physical therapies based on traditional Chinese medicine, such as spinal manipulative therapy and acupuncture (26, 27). Thus, China had the largest percentage decrease in age-standardized prevalence estimates of LBP between 1990 and 2020 (28). These countries, recognized for their substantial research output and citation impact, are home to leading institutions such as the University of Pennsylvania, Zhejiang University, and the University of Bern, which play pivotal roles in advancing this field (29).

Notably, a decline in publications count was found between 2021 and 2024. This decrease may be due to unstable therapeutic effects of MSCs caused by complex microenvironment in the damaged intervertebral disc and potential safety concerns (30). Stem cell-derived exosomes have recently gained significant research attention as a promising alternative approach. These nanovesicles exhibit comparable therapeutic properties to mesenchymal stem cells while circumventing critical limitations such as immunogenicity, pathogen transmission risks, and uncontrolled differentiation (31, 32). This shift suggests that future studies should pay more attention to explore and harness the therapeutic potentials of exosomes in IVDD.

Key journals, including *Biomaterials* and *Spine*, dominate the publication landscape, reflecting their importance in MSC and IVDD research. Notably, *Biomaterials* had an Impact Factor of 12.8 in 2023, focus on the application of MSC and biomaterials in treating IVDD, with emphasis on nucleus pulposus regeneration (33), anti-inflammatory effects (34), and prevention of calcification (35).

Authors such as Daisuke Sakai and Sibylle Grad have made significant contributions, ranking among the highest in publication output and citation impact. Dr. Daisuke Sakai notably advanced the understanding of the superior regenerative effects of nucleus pulposus cell (NCP)-derived extracellular vesicles (EVs), particularly Tie2-enhanced EVs, in treating IVDD and alleviating pain (36). Dr. Sebastian Wangler made significant contributions to highlight MSCs’ potential for tailored regenerative therapies for IVDD (37). Notably, collaboration exists between these two authors, further strengthening advancements in the field (22, 38, 39).

### Research hotspots and Frontiers

Based on the provided keyword co-occurrence analysis, research around 2014 primarily focused on foundational biological mechanisms, with particular emphasis on “gene expression,” “stromal cells,” and “articular cartilage.” Dysregulated expression of inflammatory cytokines—such as IL-1β, IL-6, and TNF-*α*—has been shown to disrupt microenvironmental homeostasis in intervertebral disc degeneration (IVDD), promoting cell senescence and apoptosis, and ultimately accelerating disease progression (40). The involvement of microRNAs (miRNAs) in IVDD has also emerged as a prominent research focus. Numerous miRNAs, including miR-138-5p, miR-10b, and miR-27b, have been implicated in regulating processes such as nucleus pulposus cell apoptosis, aberrant cell proliferation, inflammatory responses, and annulus fibrosus degeneration. These miRNAs are increasingly recognized as potential biomarkers for the diagnosis and prognosis of IVDD (41). Another key area of interest lies in the role of the extracellular matrix (ECM) in maintaining the structural and functional integrity of intervertebral discs. The ECM and intervertebral disc cells constitute a coordinated functional unit (42). During the progression of IVDD and aging, ECM components undergo significant loss and structural degradation, while the accumulation of catabolic byproducts further exacerbates the deterioration of the disc microenvironment. Immunohistochemical, gene expression, and time-lapse analyses have demonstrated that ECM architecture and collagen fiber orientation can significantly influence MSC migration within the intervertebral disc region, providing a theoretical basis for the development of tissue-engineering strategies to restore or regenerate degenerated discs (43).

Between 2016 and 2018, increasing research attention was directed toward the regenerative potential of MSC in intervertebral disc repair. Studies during this period explored how MSC interact with “chondrocytes” and contribute to the repair of articular cartilage, laying the foundation for their therapeutic applications in degenerative diseases. MSCs isolated from bone marrow (BM-MSCs), adipose tissue (AD-MSCs) and umbilical cord (UC-MSCs) show considerable promise for use in IVDD repair (44). Minting Yuan et al. demonstrated that NCP-derived acellular matrix can induce MSC differentiation into nucleus pulposus-like cells and chondrocytes, enhancing matrix production and improving hydration levels, glycosaminoglycan content, and type II collagen deposition in degenerated discs, underscoring its potential in disc regeneration therapy (45). The clinical evidence of an intradiscal therapy involving MSCs has been provided by Kumar et al. 60% of individuals displayed a significant improvement of pain, disability, and quality of life (46). However, despite the encouraging outcomes, there are many difficulties that remain prior to the MSCs-based therapies in clinical applications. The biggest obstacle is the harsh microenvironment such as hypoxia, nutrition deprivation, acidic conditions, excessive cyclic tension, high osmolarity, and inflammatory factors during the disc degeneration (47). These hostile conditions can severely impair the viability, function, and regenerative potential of MSCs, limiting the effectiveness of biologic therapies during disc regeneration.

By 2020, research emphasis began to shift toward translational and application-oriented strategies, with increasing focus on keywords such as “delivery” and “bone-marrow-cells.” Investigators explored various *in vivo* delivery systems to enhance MSC survival and regenerative efficacy. For instance, methacrylated gellan gum-based hydrogels were shown to safely encapsulate MSCs and nasal chondrocytes, supporting cell viability and chondrogenesis, and demonstrating promise for nucleus pulposus tissue engineering (48). Concurrently, there was a growing interest in enhancing the regenerative capacity of MSC through innovative approaches such as hyaluronic acid (HA) delivery systems (49, 50). Specifically, CD44, the best-characterized HA receptor, mediates migration of MSCs through interaction with extracellular HA, and its expression on activated lymphocytes aids in the primary rolling to the inflammatory sites in a HA-dependent manner (51).

Moreover, traditional scaffolds or simple biomaterials often lack the bioactivity required to modulate cellular behavior or direct stem cell differentiation. As a result, the use of biologically active, factor-loaded biomaterials has emerged as a promising strategy to improve therapeutic outcomes. For example, a Kartogenin@PLGA-GelMA/PRP composite hydrogel was designed to protect MSCs from oxidative stress within the degenerative disc microenvironment by activating the Nrf2/TXNIP/NLRP3 signaling axis. This system not only promoted differentiation into a nucleus pulposus-like phenotype but also maintained disc tissue integrity and enhanced the synthesis of nucleus pulposus-like extracellular matrix, underscoring its potential in stem cell-based therapies for IVDD (52). Additionally, platelet-rich plasma (PRP) has been investigated for its capacity to synergistically enhance MSC-mediated analgesia and tissue repair in IVDD (53). Collectively, these advancements reflect a progressive shift in research priorities toward optimizing MSC therapies through the integration of advanced biomaterials and targeted delivery platforms.

### Future research trends

Looking ahead, an analysis of recent trends, including persistent citation bursts from 2019 extending through 2024, reveals key areas likely to dominate future research in MSC and IVDD degeneration:

“Activation” of MSC and their associated signaling pathways is anticipated to remain a central research focus. Efforts to identify mechanism of improved IVDD by MSCs will likely play a pivotal role in identification of potential therapeutic targets. For example, Guo et al. demonstrated that urine-derived stem cells ameliorated IVDD by antisenescence effects and promotes NPC proliferation and ECM synthesis by activating TGF-*β* pathway (54). Cui et al. indicated that MSC-derived EVs carrying miR-129-5p confer protection against IVDD by targeting LRG1 and suppressing the activation of p38 MAPK signaling pathway, offering a novel therapeutic strategy in IVDD (55). Notably, these results suggest the EVs shuttled by MSCs can deliver exogenous microRNAs to reduce the progression of IVDD.

Despite the promises and benefits, there are still many challenges facing MSC applications for IVDD, such as their variability, scalability, and delivery, as well as ethical and safety issues (56). Recently, MSC-derived “EVs” (MSC-EVs), preventing safety concerns associated with MSC therapy, have emerged as novel drug and gene delivery tools with good therapeutic effects against IVDD. For instance, it was reported that miR-155-5p in MSC-EVs could enhance proliferation and migration, attenuate “apoptosis,” and modulate ECM secretion in chondrocytes (57). After intradiscal injection of MSC-EVs containing miR-105-5p in an animal IVDD model, NPCs “apoptosis,” senescence, and IVDD were significantly alleviated (58). Intramuscular injection of MSC-EVs also alleviates IVDD in rat models via AKT/mTOR-mediated “autophagy” (59). Additionally, “oxidative stress” is also extensively presented in IVDD. Advanced glycation end products (AGEs) usually accumulate in aging and degenerative tissues, especially in collagen-rich ones, and are closely associated with endoplasmic reticulum (ER) stress and apoptosis (60). MSC-EVs can reduce NPC AGE-induced oxidative stress and inhibit excessive activation of the unfolded protein response (UPR), subsequently alleviating apoptosis via AKT and ERK pathways (61).

Taken together, “EVs” represent a rapidly growing frontier in this field. Although EVs represent a more accessible and cell-free therapy, some challenges should be taken into consideration before translation to clinical applications such as production, safety, long-term durability, and regulation (62). First, a standardized separation process is still lacking, leading to uncertainty about EVs biological effects and safety; Second, the rapid clearance and disruption of EVs are two major challenges during applying MSC-EVs in IVDD; Third, there are still no standard techniques established for the clinical-grade production and quality control of EV-based therapy. Future work must concentrate on devising effective strategies to mitigate these challenges.

### Strengths and limitations

This bibliometric study provides a comprehensive analysis of publication trends, influential contributors, and key research focuses in MSC therapy for IVDD from 2000 to 2024. A major strength of this study lies in its multi-decade scope, which captures the evolution of foundational research to emerging frontiers in regenerative medicine. However, similar to other bibliometric studies, it has limitations. The reliance on citation metrics may not fully capture the clinical applicability or real-world impact of individual studies. Additionally, restricting the analysis to English-language publications may overlook significant contributions in other languages, potentially narrowing the scope of the findings.

## Conclusion

This bibliometric analysis reviewed 931 articles, revealing an overall upward trend in publication activity. Leading authors included Sakai Daisuke, Grad Sibylle, and Alini Mauro. China and the USA led in publication volume and citation counts. The University of Pennsylvania and Zhejiang University were the most productive institutions. Key journals included *Biomaterials*, *Spine*, and *Tissue Engineering Part A*. Future research on MSC therapy for IVDD should prioritize understanding the cellular and molecular mechanisms underlying regeneration, particularly the roles of processes such as autophagy, apoptosis, and oxidative stress in the degenerated disc microenvironment. Further investigation into patient-specific factors, including variations in the IVD microenvironment and individual responses to MSC-based treatments, will be essential for optimizing therapeutic outcomes. Advancing cell delivery methods, leveraging EVs as cell-free therapies, and developing personalized regenerative strategies will be critical for enhancing the precision, safety, and long-term efficacy of MSC therapies, ultimately improving patient outcomes and quality of life.

## Data Availability

The original contributions presented in the study are included in the article/supplementary material, further inquiries can be directed to the corresponding authors.
